# Rituximab Followed by Belimumab Controls Severe Lupus Nephritis and Bullous Pemphigoid in Systemic Lupus Erythematosus Refractory to Several Combination Therapies

**DOI:** 10.3389/fmed.2020.553075

**Published:** 2020-10-28

**Authors:** Luca Petricca, Maria Rita Gigante, Annamaria Paglionico, Stefano Costanzi, Gisella Vischini, Clara Di Mario, Valentina Varriano, Giacomo Tanti, Barbara Tolusso, Stefano Alivernini, Giuseppe Grandaliano, Gianfranco Ferraccioli, Elisa Gremese

**Affiliations:** ^1^Division of Rheumatology, Fondazione Policlinico Universitario A. Gemelli IRCCS, Rome, Italy; ^2^Nephrology Unit, Fondazione Policlinico Universitario A. Gemelli IRCCS, Rome, Italy; ^3^Division of Rheumatology, Department of Translational Medicine and Surgery, Università Cattolica del Sacro Cuore, Rome, Italy; ^4^Nephrology Unit, Department of Translational Medicine and Surgery, Università Cattolica del Sacro Cuore, Rome, Italy

**Keywords:** lupus nephritis, bullous pemphigoid, belimumab, rituximab, sequential therapy

## Abstract

Systemic lupus erythematosus (SLE) and bullous pemphigoid (BP) are chronic autoimmune diseases in which B cells play an important pathogenic role in the different stages of the disease. B cell-targeted therapies have been suggested as a new rational approach for treating SLE. Rituximab (RTX), an anti-CD20 chimeric monoclonal antibody, failed to achieve primary endpoints in two clinical trials (EXPLORER and LUNAR) despite multiple observational and retrospective studies showing its beneficial effect on SLE. Moreover, RTX is recommended in cases of BP that is unresponsive to conventional treatments. Belimumab (BLM), a human immunoglobulin G1 λ monoclonal antibody that inhibits soluble B-lymphocyte stimulator (BlyS)/B-cell activating factor (BAFF), is the only biological treatment approved for standard therapy of refractory autoantibody-positive active SLE. Animal models and a few case reports have supported the efficacy of the combined use of RTX followed by BLM as maintenance therapy in severe lupus nephritis (LN), suggesting that their combined use may be more effective than their single use, without compromising safety. In this study, we describe the clinical case of a SLE patient with predominant renal involvement in overlap with BP, refractory to conventional therapy including RTX alone, achieving significant steroid sparing and clinical remission under sequential treatment of RTX-BLM. Moreover, we describe the first case of BP successfully treated with BLM. This case report may encourage further clinical research studies in B lymphocyte targeted combination therapy in patients affected by SLE with major organ involvement or with refractory disease, suggesting that RTX and BLM sequential therapy may be a valid option for the treatment of SLE manifestations, including conventional therapy and RTX-resistant LN.

## Introduction

Systemic lupus erythematosus (SLE) and bullous pemphigoid (BP) are chronic autoimmune diseases in which B lymphocytes play a primary pathogenic role as they are implicated in the induction and progression of these diseases (1, 2). Only a few cases of patients affected by SLE in overlap with BP have been described in the literature (3–5). B cells exert their pathogenic action not only by producing autoantibodies but also by presenting autoantigens to T lymphocytes and secreting of a wide variety of proinflammatory cytokines, thus perpetuating the activation of the immune system (6). Rituximab (RTX), a chimeric monoclonal antibody that targets CD20 antigen on B cells, is successfully used to treat various autoimmune diseases by depleting B lymphocytes. Although some observational and retrospective studies have shown beneficial effects of RTX in SLE patients (7, 8), it failed to achieve the primary endpoints in the EXPLORER and LUNAR trials (9, 10), probably due to a wrong trial design. Moreover, RTX has been shown to be effective in BP patients who were unresponsive or with unacceptable side effects to conventional immunosuppressive drugs (11–15). However, the position of RTX within the therapeutic flowchart of SLE and BP diseases is still unknown. Belimumab (BLM) is a human immunoglobulin G1 λ monoclonal antibody that inhibits soluble B-lymphocyte stimulator (BlyS)/B-cell activating factor (BAFF) (16), and in 2011, BLM was approved for the treatment of standard therapy-refractory autoantibody-positive active SLE (17, 18). Moreover, BLM has been proven to be effective to treat moderate SLE with skin, articular, and hematologic abnormalities (19), although it is not licensed to treat severe lupus nephritis (LN) (20–22). To date, sequential therapeutic schemes of RTX followed by BLM have not been well-studied. Animal models (23) and few case reports support the efficacy of the combined use of RTX followed by BLM as maintenance therapy in severe LN (24–27), suggesting that their combined use may be more effective than their single use, without compromising safety. In this study, we reported the clinical case of a SLE patient with predominant renal involvement in overlap with BP, refractory to conventional therapy including RTX alone, achieving significant steroid sparing and clinical remission under sequential treatment of RTX-BLM. Moreover, we describe here the first case of BP successfully treated with BLM.

## Case Presentation

**Figure 1:**
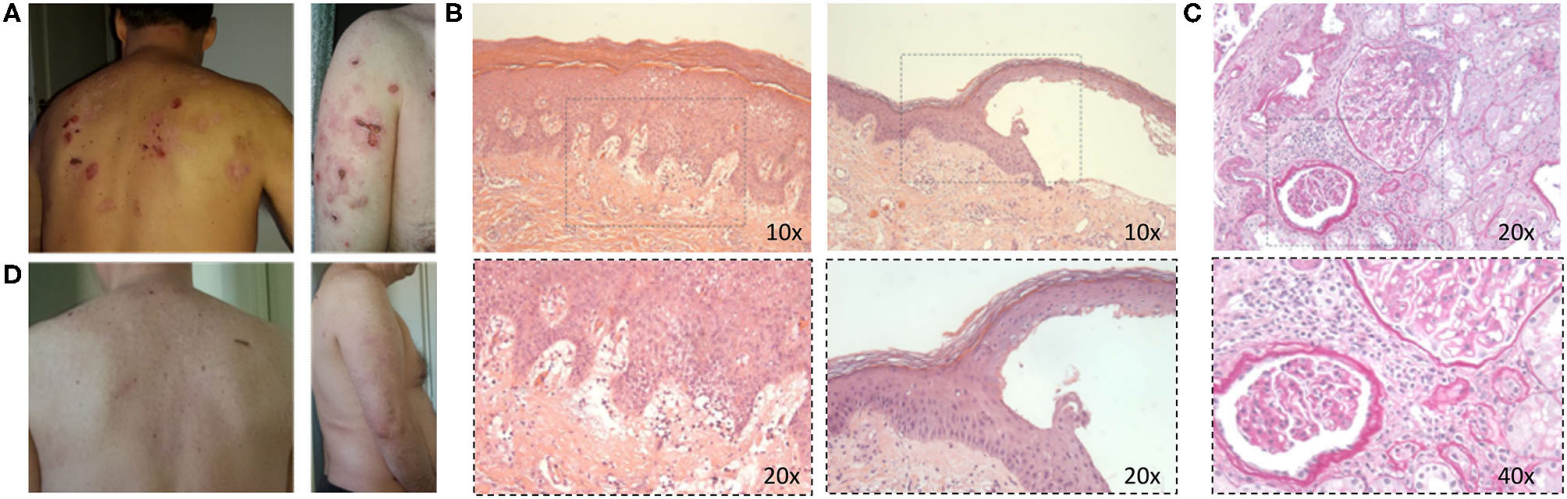
**(A)** Active bullous pemphigoid skin lesions. **(B)** Histological images in hematoxylin and eosin staining of skin lesion biopsy showing subepidermal blister with a mixed inflammatory infiltrate rich in eosinophils mostly localized in a superficial dermis (10× and 20× magnification, respectively). **(C)** Periodic acid–Schiff staining images of kidney biopsy showing mesangial hypercellularity; the vessels show a slight moderate reduction of the lumen for sclerosis and myointimal hyperplasia, and vasculitic aspects are not observed (20× and 40× magnification, respectively). **(D)** Improvement of skin manifestations after therapy.

**Figure 2:**
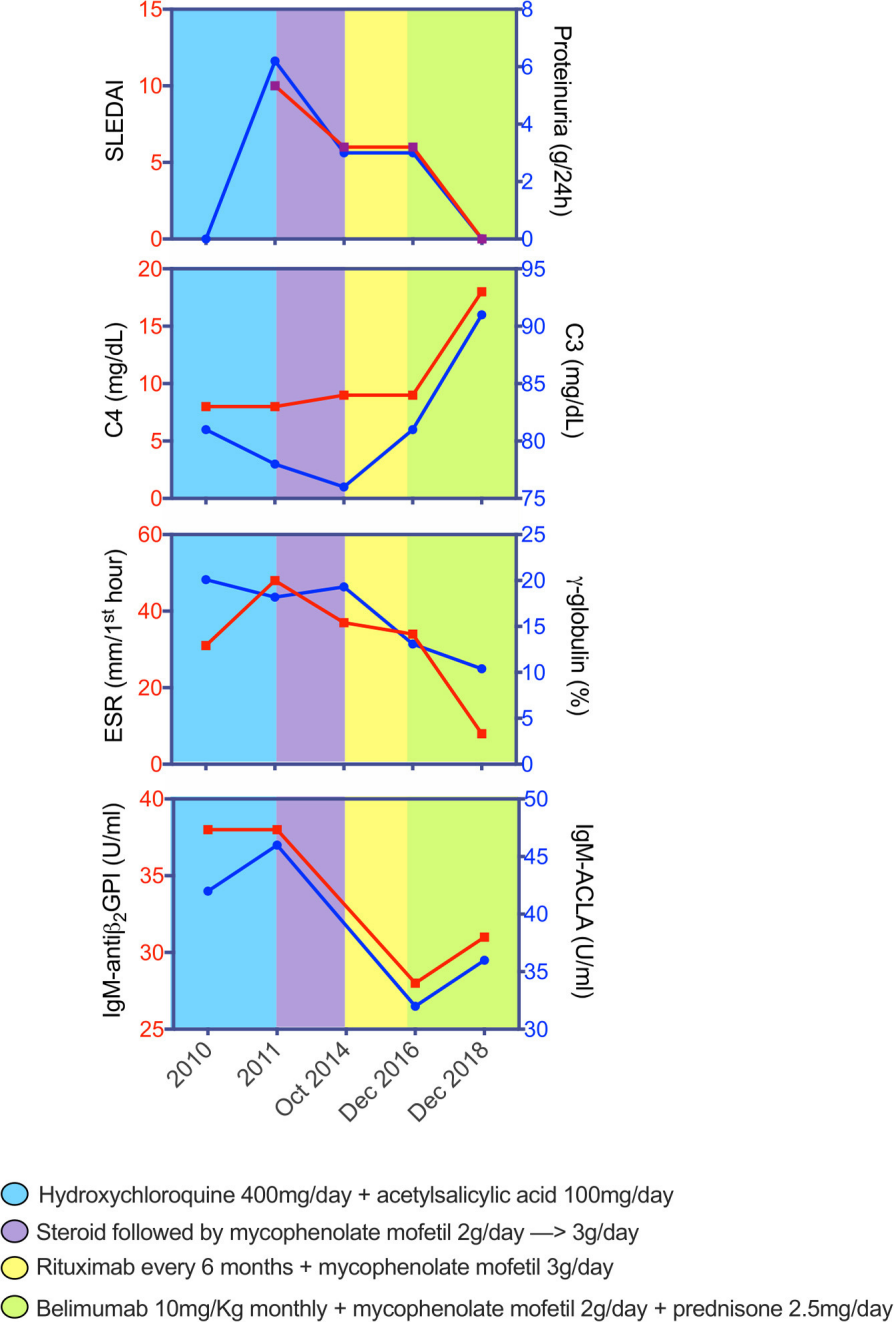
Changes of SLEDAI, proteinuria, and blood parameters across disease course of patient with systemic lupus erythematosus and bullous pemphigoid. Graphs depict the dynamics of each parameter while receiving different therapeutic schemes. SLEDAI, Systemic Lupus Erythematosus Disease Activity Index; C3, fraction 3 of the complement; C4, fraction 4 of the complement; ESR, erythrocyte sedimentation rate; ACLA, anti-cardiolipin antibodies; antiβ2-GPI, anti-β2 Glycoprotein 1 antibodies.

We describe the clinical case of a 51-year-old Italian man who was diagnosed as having Undifferentiated Connective Tissue Disease in 2010 because of the presence of Raynaud's phenomenon, arthralgias, positivity for antinuclear antibody (ANA, 1:160 fine speckled), antiphospholipid antibodies (aPL) [(anticardiolipin antibodies (ACLA) IgM, 42 U/ml (normal range <20 U/ml), and anti-β2 Glycoprotein 1 (antiB2GP1) IgM, 38 U/ml (normal range <20 U/ml)], and a mild hypocomplementemia, C3 81 mg/dl (normal range 90–180 mg/dl) and C4 8 mg/dl (normal range 8–32 mg/dl). The patient did not report a family history of rheumatic disorders or a personal history of comorbidities and/or previous major surgery. A treatment with hydroxychloroquine (HQC) 400 mg daily and acetylsalicylic acid 100 mg daily was started. In 2011, the patient developed diffuse bullous skin lesions and a skin biopsy of a trunk lesion showed a typical histological picture for BP. Therefore, topical and oral steroid (0.25 mg/kg daily) therapy was started. Subsequently, the patient developed periorbital and lower limb edema, with proteinuria (6.2 g/daily), and a renal biopsy was performed showing histological findings of diffuse membranous glomerulonephritis associated with moderate mesangial hypercellularity (Class V according to ISN/RPS classification, 2003) (28). Therefore, a diagnosis of SLE was made [due to the presence of nephritis, arthritis, ANA/aPL positivity (ACLA IgM 46 U/ml and antiB2GP1 IgM 38 U/ml), and complement level reduction (C3 78 mg/dl and C4 8 mg/dl)] with a Systemic Lupus Erythematosus Disease Activity Index (SLEDAI) of 10; intravenous therapy with steroid (methyl-prednisolone pulses 250 mg for three consecutive days) was started followed by oral prednisone 1 mg/kg daily, subsequently tapered, and combined with mycophenolate mofetil (MMF) 2,000 mg daily and angiotensin-converting enzyme inhibitors, obtaining renal and cutaneous remission. In 2013, after steroid discontinuation, the patient experienced a proteinuric flare (2.5 g/daily) and a BP exacerbation. A second renal biopsy was performed confirming the previous histological nephritis class, and the repetition of skin biopsy documented a histological picture of leukocytoclastic vasculitis in overlap with BP. Oral prednisone was restarted at a dose of 0.5 mg/kg daily, with a slow tapering, and MMF dose was increased at 3,000 mg daily, reaching a resolution of the skin manifestations. In 2014, because of a further proteinuric flare (3 g/daily) and BP skin lesions worsening, B cells depletive treatment with RTX (two infusions of 1 g 14 days apart) was administered in association with MMF (3,000 mg daily) with a partial and temporary clinical remission. Therefore, therapy with Tacrolimus (5 mg/daily) in association with MMF was started, but quickly stopped because of a facial cutaneous rash, and followed by a combination treatment of MMF and Cyclosporine (200 mg/daily), discontinued for the same adverse event. In December 2015 and July 2016, two further retreatments with RTX were performed, with partial clinical response. In December 2016, because of the persistence of hypocomplementemia (C3 81 mg/dl, C4 9 mg/dl), proteinuria (3 g/daily) (SLEDAI 6), and BP lesions, the patient repeated skin and kidney biopsy, showing persistence of skin and kidney active inflammation (Figure 1). Therefore, RTX was discontinued and intravenous BLM (SLE therapeutic scheme, 10 mg/kg monthly) in association with MMF 2,000 mg daily and low-dose prednisone (10 mg daily) was started, leading to a progressive improvement of both renal and skin manifestations. At 24 weeks of follow up, the patient showed a complete cutaneous remission and a significant reduction until normalization of proteinuria, maintaining a complete clinical remission (SLEDAI 0) up to 52 weeks of follow up, allowing a significant reduction of prednisone dosage to 2.5 mg/daily. At the last clinical assessment, proteinuria was absent with normal complement levels (C3 91 mg/dl, C4 18 mg/dl) (Figure 2). Nowadays, the patient is continuing therapy with BLM and MMF and with low dose (2.5 mg/daily) of prednisone without further SLE and BP flares.

## Discussion

The patient described in this clinical case had long-standing relapsing LN and BP, with skin and kidney biopsies showing persistent tissue inflammation. He had been treated with many different schemes, all of them with unsuccessful outcomes. Renal and skin flares were repeatedly treated with RTX, a drug frequently effective in this clinical setting (29). However, in the patient we describe, RTX only helped to achieve partial remission with early relapses. Thus, BLM was used as an additional option. To date, BLM has been used in patients with LN, mainly to maintain remission (25, 30); however, some clinical cases have been described reporting the use of BLM in the treatment of multiple therapy refractory patients with LN (31). Awaiting the results of ongoing clinical trials, we believe that BLM could be added to the list of potential treatments for patients with refractory LN. It is established that B cell hyperactivity is a landmark in SLE (32), and autoantibody positivity significantly characterizes patients with LN. The randomized trials BLISS-52 and BLISS-76 have documented the efficacy of BLM in autoantibody-positive SLE without previous RTX exposure and without major organ involvement (17, 18), and there is a randomized clinical trial demonstrating that BLM plus Standard Therapy (ST) significantly improves LN renal responses compared with ST alone (BLISS-LN trials; Clinicaltrials.gov identifier: NCT01639339; GSK study 114054) (33). RTX acts by causing a marked B cell depletion (BCD) compared to the more modest effects on BCD induced by BLM. Therefore, the rationale for their consecutive use comes from the observation that significantly higher serum BLyS levels were found during B cell repopulation after RTX treatment, thus leading to disease flares (25, 31, 32). In this context, BCD may lead to ever-increasing levels of BAFF, with an increase in anti-dsDNA antibody levels and disease flare even at low B cell rate (23, 31). Moreover, elevated BLyS plasma levels after RTX would be associated with a paradoxical proliferation of pathogenic B lymphocytes, possibly explaining the therapeutic failure of RTX in clinical trials (34). On the other hand, the increase in BLyS levels could be due to the reduction of its specific receptors that are expressed on B lymphocytes (BAFF-R) (35). Therefore, further studies are needed to address this specific issue.

Currently, two ongoing open-label clinical trials are evaluating the efficacy of sequential therapy of RTX and BLM: the CALIBRATE (Rituximab and Belimumab for Lupus Nephritis, https://clinicaltrials.gov/ct2/show/NCT02260934, 2015) and the SYNBIoSe study (Synergetic B-Cell Immunomodulation in SLE, https://clinicaltrials.gov/ct2/show/NCT02284984, 2015), respectively. Therefore, the rationale for the combination therapy of BLM with RTX could be to operate through complementary and synergistic mechanisms, as demonstrated in preclinical studies in a lupus-prone mouse model (34, 36). BLM induces the mobilization of memory B lymphocytes from tissues despite an overall reduction in peripheral B cells (37). This phenomenon may lead tissue-resident B cells to be more susceptible to depletion by RTX. Moreover, inhibition of high serum BLyS levels could have favorable quantitative and qualitative effects on the reconstitution of B cells after BCD (32). Recently, a randomized, double-blind, placebo-controlled, 104-weeks superiority study (BLISS-BELIEVE) was started, whose objective is to evaluate the efficacy, safety, and tolerability of a combination therapy with subcutaneous BLM and a single cycle of RTX (1,000 mg at weeks 4 and 6 from the beginning of BLM) compared with BLM alone in adult SLE patients (38). In a phase 2, open-label, single-arm proof-of-concept study conducted by Kraaij et al. (39), SLE patients with severe and refractory disease were treated with a combination of RTX (1000 mg at weeks 0 and 2) and BLM (10 mg/kg BLM at weeks 4, 6, 8, and then every 4 weeks) where an increase of BlyS levels upon RTX-mediated BCD was observed, repealed by subsequent BLM treatment, leading to ANA reduction and to regression of excessive NET formation (neutrophil extracellular traps, web-like structures composed of chromatin backbones and granular molecules, released by activated neutrophils through a process called “NETosis”), with a reduction of proteinuria, SLEDAI, and steroid doses.

In conclusion, our clinical case suggests that the RTX and BLM combination therapeutic scheme appears to be safe and successful in achieving a clinically significant response, thus representing a valid option for the treatment of severe SLE manifestations, including LN resistant to conventional therapy and RTX. Moreover, to our knowledge, this is the first case of BP described in the literature successfully treated with BLM.

## Data Availability Statement

The original contributions presented in the study are included in the article/supplementary material, further inquiries can be directed to the corresponding author/s.

## Ethics Statement

Written informed consent was obtained from the patient for the publication of any potentially identifiable images or data included in this article.

## Author Contributions

All authors listed have made a substantial, direct and intellectual contribution to the work, and approved it for publication.

## Conflict of Interest

The authors declare that the research was conducted in the absence of any commercial or financial relationships that could be construed as a potential conflict of interest.
